# Chronic circadian misalignment accelerates sarcopenia progression in mice

**DOI:** 10.3389/fphys.2025.1686942

**Published:** 2025-11-13

**Authors:** Takashi Seya, Nobuya Koike, Naoki Okubo, Yasuhiro Umemura, Yoshiki Tsuchiya, Kazuya Yabumoto, Yasuhiro Endo, Kanako Iinuma, Akiyo Kakibuchi, Akira Sugimoto, Kenji Takahashi, Seung-Hee Yoo, Zheng Chen, Kazuhiro Yagita

**Affiliations:** 1 Department of Physiology and Systems Bioscience, Kyoto Prefectural University of Medicine, Kyoto, Japan; 2 Department of Orthopaedics, Graduate School of Medical Science, Kyoto Prefectural University of Medicine, Kyoto, Japan; 3 Department of Biochemistry and Molecular Biology, The University of Texas Health Science Center at Houston, Houston, TX, United States

**Keywords:** circadian rhythm, circadian misalignment, sarcopenia, frailty, skeletal muscle, TWEAK/Fn14 signaling

## Abstract

**Introduction:**

In today’s 24/7 society, circadian misalignment caused by environmental and lifestyle factors is associated with various adverse health consequences. Understanding tissue-specific pathology is required to counter this growing public health challenge. A potential association of environmental circadian misalignment with sarcopenia, or accelerated loss of skeletal muscle strength and mass, is poorly documented.

**Methods:**

14-week-old wild-type C57BL/6J male mice were exposed to a chronic jet lag (CJL) paradigm consisting of an 8-h phase advance every 4 days (the ADV group) or a fixed light-dark cycle (the LD group) for 64 weeks. Grip strength was measured during the experiment, and hindlimb muscle weight was assessed after the 64-week CJL. In addition, transcriptomic and histological analysis of the hindlimb muscles were performed in all animals.

**Results:**

ADV mice exhibited significant reductions in grip strength and muscle weight relative to LD mice. Transcriptomic and histological analyses showed activation of TWEAK/Fn14 signaling and reduced myofiber cross-sectional area, hallmark features of sarcopenia, in the ADV group. Somewhat surprisingly, ADV mice showed increased centrally nucleated fibers, myosin heavy chain co-expressing fibers, and myogenic gene expression, suggesting that compensatory muscle regeneration and remodeling processes are activated but remain insufficient to counter muscle atrophy.

**Conclusion:**

These findings demonstrate that circadian misalignment is a potential risk factor for sarcopenia, underscoring circadian rhythms as a key regulator and actionable target for sarcopenia prevention.

## 1 Introduction

The circadian clock is our biological time-keeping machinery that orchestrates myriad physiological and behavioral processes over the 24-h daily cycle. The circadian system consists of a central clock, the hypothalamic suprachiasmatic nucleus (SCN), and peripheral clocks in various tissues throughout the body, including skeletal muscle (Bollinger and Schibler, 2014; Patke et al., 2020). In coordination with environmental cycles, the SCN synchronizes peripheral clocks to regulate tissue-specific and systemic circadian rhythms to maintain physiological homeostasis (Pilorz et al., 2018). Whereas different species have distinct intrinsic circadian period lengths, their alignment with environmental cycles is essential for maintaining physiological homeostasis and survival (Eastman et al., 2015; Eckel-Mahan and Sassone-Corsi, 2015). Conversely, studies in a variety of experimental organisms have reported that a misalignment between the intrinsic circadian period and the external cycle is associated with impaired growth and reduced lifespan (Highkin and Hanson, 1954; Hurd and Ralph, 1998; Ouyang et al., 1998; Pittendrigh and Minis, 1972; Takasu et al., 2015). Likewise, in our modern society, the prevalent 24/7 lifestyles perturb the harmony between endogenous oscillations and the natural light:dark cycles, disrupting intrinsic circadian physiological rhythms. Particularly, circadian misalignment in shift workers has been associated with aggravated risk for various diseases including diabetes, metabolic syndrome, cardiovascular disease, menstrual irregularities, infertility, mood disorders, and breast and prostate cancer (Boivin et al., 2021). Since shift workers constitute approximately 20% of the world’s workforce (Ward et al., 2019), the adverse health consequences of circadian misalignment represent a critical public health crisis. It is imperative to decipher the tissue-specific consequences toward the development of efficacious interventional strategies.

Skeletal muscles are essential for movement and posture (Frontera and Ochala, 2015). At the cellular level, myofibrils within multinucleated muscle fibers contract to perform locomotor functions (Sweeney and Hammers, 2018). When myofibers are damaged by overloading, such as during resistance exercise, satellite cells surrounding the myofiber are activated and undergo myoblast proliferation and differentiation. These myoblasts then fuse with myotubes and mature into fast or slow muscle fibers, thereby maintaining skeletal muscle mass and strength (Chargé and Rudnicki, 2004). However, an imbalance between the degenerative and regenerative processes in the skeletal muscle can reduce its weight and function (Blau et al., 2015; Muñoz-Cánoves et al., 2020; Thorley et al., 2015). Aging is known to be accompanied by a progressive decline in skeletal muscle mass, strength, and function, referred to as sarcopenia (Cruz-Jentoft and Sayer, 2019). Sarcopenia is a major public health problem because it increases the risk of fractures and frailty in the elderly, leading to compromised quality of life and increased mortality (Wilson et al., 2017; Xu et al., 2021; Zhang et al., 2018). The pathophysiology of sarcopenia, however, remains incompletely understood due to its multifactorial complexity and interrelated causes, such as reduced physical activity and inadequate nutritional intake.

The circadian clock is known to play an important role in skeletal muscle physiology. While circadian functions deteriorate over aging, whether circadian dysfunction accelerates the progression of sarcopenia is unknown (Choi et al., 2019; Harfmann et al., 2015). It has been reported that the genetic disruption of core clock genes, including ablation of *Bmal1*, leads to sarcopenia like phenotypes in mice. Specifically, global *Bmal1* knockout mice exhibit severe systemic abnormalities with marked reductions in skeletal muscle mass and strength (Andrews et al., 2010; Kondratov et al., 2006). In addition, muscle-specific *Bmal1* knockout mice show reductions in muscle mass and function (Fernández-Martínez et al., 2024), suggesting that both systemic and muscle intrinsic circadian disruptions can contribute to sarcopenia like changes. However, these genetic models may not adequately recapitulate human pathophysiology of sarcopenia, given the various confounding factors including lifestyle, activity level, and dietary habits (Choi et al., 2019; Palmese et al., 2025). Therefore, it is necessary to establish an experimental animal model that captures key etiological processes relevant to human disease and aging. Previously, we established a long-term circadian misalignment cohort model using wild-type C57BL/6 mice subjected to a chronic jet-lag (CJL) paradigm mimicking shift work. We discovered that chronic circadian misalignment, specifically the phase advance condition (ADV) described herein, exacerbates immune senescence, chronic inflammation, mortality, and non-alcoholic steatohepatitis of the liver (Inokawa et al., 2020; Koike et al., 2024).

Recognizing the paucity of evidence linking environmental circadian disruption to skeletal muscle loss in mice and humans, we leveraged the chronic circadian misalignment model over the prolonged experimental duration to investigate the relationship between circadian misalignment and sarcopenia. Wild-type C57BL/6J mice were exposed to the CJL of 8-h phase advance every 4 days (the ADV condition) for 64 weeks or the fixed light-dark cycle (the LD condition). The results showed that the ADV group displayed significantly lower grip strength and normalized muscle weight than the 12-week-old young mice and the LD control group, indicating that chronic circadian misalignment accelerated the progression of sarcopenia with reduced muscle weight and strength. Transcriptome and histological analysis under the CJL condition revealed activation of the TWEAK/Fn14 signaling pathway and reduced myofiber cross-sectional area (CSA), suggesting that this mouse model recapitulates the pathophysiology of sarcopenia. Furthermore, we observed enhancing expression of myogenic regulators and an increase in centrally nucleated fibers and myosin heavy chain (MHC) co-expressing fibers in ADV mice, suggesting that a compensatory muscle regeneration under the circadian misalignment condition was insufficient to prevent muscle atrophy. These results suggest a close link between circadian misalignment and the pathogenesis of sarcopenia, and provide evidence that circadian rhythms represent an interventional target for sarcopenia.

## 2 Materials and methods

### 2.1 Mice

Male C57BL/6JJcl mice (10 weeks old) were purchased from CLEA Japan, Inc. (Tokyo, Japan). Mice were randomly assigned to LD and ADV groups (n = 16) and housed in individual cages. The group was housed (170 × 350 × 145 mm) with a 120-mm diameter running wheel (SANKO, Osaka, Japan). All mice were kept in light-shielded mouse housing boxes at the room temperature of 25.0 °C ± 1.5 °C with food and water available *ad libitum*, as described previously (Koike et al., 2024). The mice were entrained to a 12:12-h light-dark cycle with an 8:00–20:00 light period for 2 weeks, followed by 2 weeks in the constant darkness (DD). After DD exposure, mice were returned to the normal light condition (8:00–20:00) followed by the same LD condition (light–dark condition with an 8:00–20:00 light period) or the ADV condition (8-h phase advance once every 4 days) for 64 weeks. Body weight of each mouse was measured once every 2 weeks during these CJL conditions, and relative body weight was calculated by comparing each mouse’s weight at the time of purchase (10 weeks old). Food intake was measured by providing a pre-weighted food pellet (CRF-1; Oriental Yeast Co., Ltd., Tokyo, Japan) in the top hopper of each home and weighing the remaining food once every 2 weeks. Criteria for humane endpoints established at the beginning of the study included: exhibiting gait abnormalities or showing weight below 20% of what is expected for the animal. Two mice in the LD group were excluded from analysis, as one exhibited gait abnormalities due to injuries was euthanized (4% isoflurane inhalation followed by cervical dislocation), and the other was found dead. Tissue sampling from LD (n = 14), ADV (n = 16) mice was performed from 9:30 to 17:30 after 64 weeks under the CJL paradigm, when ADV mice were in the same light-dark cycle as LD mice with an 8:00–20:00 light period. Young mice were evaluated separately as a control group. At 10 weeks of age, the mice (n = 30) entrained to the same 12:12-h light-dark cycle protocol as the experimental groups for 2 weeks. The sample size for the young group was calculated based on grip strength data from the LD and ADV groups using a power analysis (α = 0.05, power = 0.8). After this entrainment period, body weight, food intake, and grip strength were measured. For tissue collection, mice were anesthetized with 4% isoflurane inhalation and blood was collected from the heart. Then the mice were decapitated, and the gastrocnemius, soleus, and tibialis anterior muscles were collected. The muscle weights were measured using an electronic analytical balance (ER-180A, A&D Company, Tokyo, Japan). The plantaris muscle shares a similar fiber type composition with the gastrocnemius, making it difficult to separate the two. Therefore, it was collected together as a part of gastrocnemius muscle (Adamovich et al., 2021). Muscle weights were determined as the average of both legs and normalized to body weight at the time of sampling (Shang et al., 2020). After weighing, for RNA-seq analysis, the gastrocnemius muscle was incubated with RNAlater (Thermo Fisher Scientific) for several hours according to the manufacturer’s instructions and snap-frozen in liquid nitrogen. For histological analysis, the soleus muscle was fixed in 10% neutral buffered formalin. All experiments were approved by the Experimental Animals Committee, Kyoto Prefectural University of Medicine (approval No. M2022-194-1, M2023-191, M2024-180-1), and were performed in accordance with the institutional guidelines and Guidelines for Proper Conduct of Animal Experiments by the Science Council of Japan.

### 2.2 Behavioral analysis

The behavioral analysis of the mice exposed to the CJL conditions was performed as described in our previous reports (Inokawa et al., 2020; Koike et al., 2024). The wheel-running frequency was measured by counting the number of signals from a magnet sensor (59070-010, Littelfuse Inc., Chicago, IL, United States). Clocklab software (Actimetrics, Wilmette, IL, United States) was used to analyze the behavioral activity in wheel revolutions per 1-min bin collected using CompACT AMS (Muromachi Kikai Co. Ltd., Tokyo, Japan) and ClockLab data collection (Actimetrics, Wilmette, IL, US). The mean activities every 4 weeks were calculated using the daily activities. Relative activity was calculated based on activity levels during the 3 weeks prior to the start of the CJL condition (Koike et al., 2024). Chi-square periodogram analysis was carried out in R using the Rethomics package on 14-day activity segments (Geissmann et al., 2019).

### 2.3 Four-limb grip strength test

The four-limb grip strength test was performed with the timing of light conditions aligned between the LD and ADV groups, using a digital force meter (GPM-101B; Melquest, Toyama, Japan). Mice gripped a metal net with all four limbs, and their tails were gently pulled backward in a horizontal direction. The maximum grip force exerted when the mice released the net was recorded as the grip strength. Each mouse underwent the test three times, and the average of the three measurements was recorded as the final grip strength (Shang et al., 2020).

### 2.4 Histological analysis

The gastrocnemius and soleus muscles were fixed in 10% neutral buffered formalin at room temperature for 2 days. Haematoxylin and eosin (HE) staining and immunohistochemical staining were performed by KAC Co., Ltd. (Kyoto, Japan). To identify MHC co-expressing fibers, immunohistochemical staining for fast and slow MHC was performed on serial sections, and corresponding fibers were identified by positional matching across adjacent sections. Monoclonal anti-fast MHC antibody (M4276, Sigma-Aldrich) and anti-slow MHC antibody (ab234431, Abcam) were used, respectively. Images of all muscle fibers were obtained with a BZ-X710 microscope (Keyence, Osaka, Japan). The CSA of the gastrocnemius and soleus muscles was calculated from the entire HE-stained sections using the BZ-X700 Analyzer (Keyence, Osaka, Japan). In the gastrocnemius muscle, fiber size distribution and mean CSA were quantified by measuring at least 100 fibers from HE-stained sections (Wang et al., 2018). Centrally nucleated fibers were defined as fibers with nuclei not in contact with the sarcolemma (Kay et al., 2022), and their number was normalized to muscle CSA. In the soleus muscle, the number of MHC co-expressing fibers was determined from immunohistochemically stained sections. For each sample, the average of three sections was used for quantification, and values were normalized to muscle CSA. Centrally nucleated fibers were also counted on the same immunostained sections.

### 2.5 RNA-seq

Frozen gastrocnemius muscle was homogenized twice for 30 s in TRIzol reagent (Thermo Fischer Scientific) with 5-mm- and 3-mm-diameter stainless beads using a desktop bead Crusher Shakeman 6 (Bio Medical Science Inc., Tokyo, Japan) at 3,500 rpm. Poly (A)-enriched stranded RNA sequencing was carried out by the NGS core facility at the Research Institute for Microbial Diseases of Osaka University on Illumina NovaSeq X with 101-bp paired-end reads. After adaptor sequences were trimmed using Trimmomatic, the sequence reads were mapped to the mouse genome (GRCm39/mm39) using STAR as described previously (Bolger et al., 2014; Dobin et al., 2013; Koike et al., 2024). To obtain reliable alignments, the reads with a mapping quality of less than 10 were removed by SAM tools (Li et al., 2009). The University of California, Santa Cruz (UCSC) known canonical gene set (57,186) were used for annotation, and the reads mapped to the exons were quantified using analyzeRNA.pl script in the Homer software with an option of–rpkm or -noadj for FPKM or raw read counts, respectively (Heinz et al., 2010). Since the Homer treat each half of the read separately and count each as 0.5 reads for paired-end reads, the raw read counts were rounded to the nearest integer before transforming rlog using DESeq2 (Love et al., 2014). To report one isoform per locus (gene symbol), the longest expressed isoform was chosen. We assumed that a gene was expressed if there were more than 20 reads mapped on average in the exons of the gene. The expression level cutoff, average FPKM >0.5, was used for the downstream data analysis. Differentially expressed genes in the RNA-seq data were determined using DESeq2 with thresholds of false discovery rate (FDR) < 0.05 and fold change >1.3. Gene ontology enrichment analysis of differentially gene expressed genes and gene set enrichment analysis was carried out using clusterprofiler in R language (Yu et al., 2012) with a threshold of false discovery rate (FDR) < 0.05. The enriched GO terms with less than 5 gene counts were excluded.

### 2.6 Statistical analysis

Data are presented as mean ± SD. Statistical analysis was performed by the statistical methods stated in each legend using GraphPad Prism version 10.0 or R software. The significance level was set at p < 0.05 unless otherwise noted.

## 3 Results

### 3.1 Circadian misalignment accelerates the progression of sarcopenia

**FIGURE 1:**
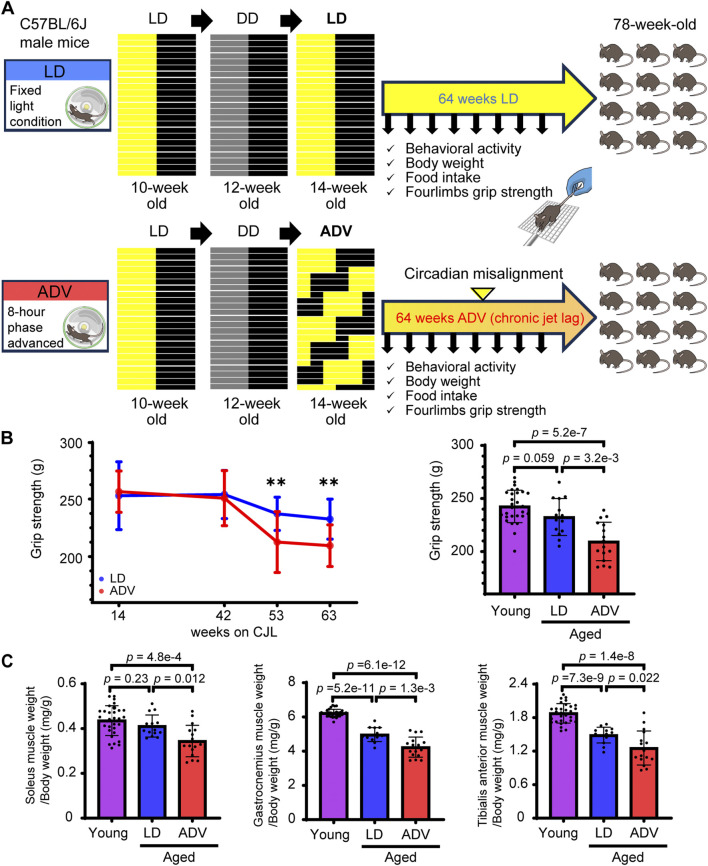
Circadian misalignment accelerates the progression of sarcopenia. **(A)** Schematic illustration of the experimental design. **(B)** Longitudinal changes in grip strength in LD and ADV groups. The grip strength was measured in young mice (12 weeks old) and in aged mice after 63 weeks on CJL. **(C)** The muscle weights normalized to body weight of soleus, gastrocnemius, and tibialis anterior muscles were measured in young mice (12 weeks old) and in aged mice after 64 weeks on CJL. Each circle represents the data from one animal. Bar plots shown are mean ± SD (Young, n = 30; LD, n = 14; ADV, n = 16). The significance was determined using two-sided Wilcoxon rank-sum test corrected by Benjamini–Hochberg multiple testing.

Wild-type C57BL/6J male mice were exposed to either the normal LD cycles or the previously established circadian misalignment paradigm, the aforementioned ADV conditions (Inokawa et al., 2020). Given that mice aged 18 months or older are commonly used as a natural aging model exhibiting early features of age-related sarcopenia, including reduced grip strength and muscle weight, we subjected 14-week-old mice to 64 weeks under the LD and ADV conditions, when all mice reached approximately 18 months of age (Shang et al., 2020; Xie et al., 2021). Before the initiation of the CJL paradigm, all mice were first entrained to a 12-h/12-h LD cycle for 2 weeks, followed by 2 weeks in constant darkness (DD) to measure baseline free-running activities (Figure 1A). In the ADV group, the circadian period progressively diverged from their intrinsic circadian rhythm with age, indicating that ADV mice underwent chronic circadian misalignment (Supplementary Figure 1). To assess whether these mice developed age-related sarcopenia based on human clinical criteria (e.g., reduced muscle mass and strength compared to younger populations) (Cruz-Jentoft et al., 2019), we evaluated grip strength and muscle weight normalized to body weight relative to young 12-week-old mice. Longitudinal assessments revealed that, after 53-week CJL treatment, ADV mice were impaired in grip strength compared to LD mice (Figure 1B). Furthermore, ADV mice exhibited significant decreases in not only grip strength, but also the normalized weights of soleus, gastrocnemius, and tibialis anterior muscles relative to both young mice and LD mice (Figures 1B,C). On the other hand, LD mice showed significant reductions in the normalized weights of gastrocnemius and tibialis anterior muscles, whereas grip strength and the normalized weight of soleus muscle were not significantly reduced compared to young mice (Figures 1B,C). ADV mice also showed increased body weight and reduced activity levels compared with LD mice, without an increase in food intake (Supplementary Figure 2), consistent with a previous report (Koike et al., 2024). These experiments using the CJL model reveal that environmental circadian misalignment accelerates the progression of sarcopenia.

### 3.2 CJL induces myogenic gene expression

**FIGURE 2:**
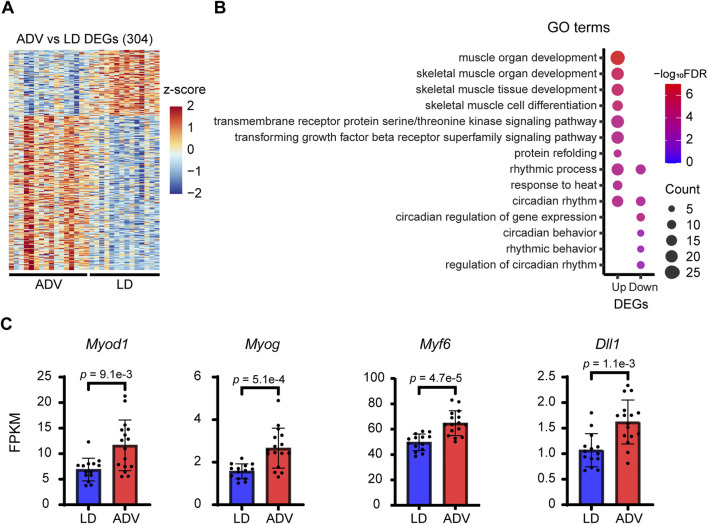
CJL induces myogenic gene expression. **(A)** Heatmap views of DEGs between LD and ADV condition in gastrocnemius muscle. A total of 304 DEGs were represented as a horizontal line ordered vertically by log2 fold changes determined by DESeq2. **(B)** Bubble plots of top 10 enriched GO terms in up- and downregulated DEGs. Color gradients ranging from red to blue correspond to in order of increasing FDR and the gene count of each GO term is shown. **(C)** FPKM expression levels of key myogenic regulatory factors. Bar plots shown are mean ± SD (LD, n = 14; ADV, n = 16). P values indicated are based on two-sided Wilcoxon rank-sum test.

To investigate the molecular basis for the distinct effects of circadian misalignment on the grip strength and muscle weight, we performed RNA-seq analysis using gastrocnemius muscles from the mice exposed to LD and ADV conditions for 64 weeks. Analysis of differentially expressed genes (DEGs) identified differential expression of 304 genes between LD and ADV groups (Figure 2A; Supplementary Table 1). GO enrichment analysis of the DEGs showed that GO terms including circadian rhythm, rhythmic process, muscle organ development, and skeletal muscle cell differentiation were significantly enriched (Figure 2B; Supplementary Table 2). These enriched GO terms included key myogenic regulators such as *Myod1*, a master transcription factor involved in myoblast proliferation and differentiation. In addition, myogenesis-related genes such as *Myog* and *Myf6*, which promote myotube formation by myoblast fusion (Aziz et al., 2010; Hasty et al., 1993), and *Dll1*, which is activated by *Myod1* and promotes the myogenic process (Kollias et al., 2006; Zhang et al., 2021), were significantly upregulated in the ADV group compared to the LD group (Figure 2C). These findings suggest that circadian misalignment may promote muscle regeneration and remodeling processes.

### 3.3 Altered expression of the TWEAK/Fn14 signaling pathway in CJL mice

**FIGURE 3:**
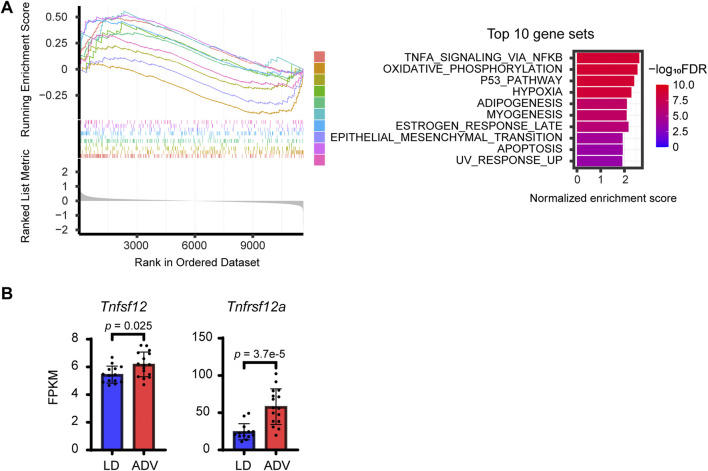
Altered expression of the TWEAK/Fn14 signaling pathway in CJL mice. **(A)** GSEA enrichment plots in the top 10 HALLMARK datasets. Normalized enrichment scores and FDR values are shown. **(B)** FPKM expression levels of *Tnfsf12* and *Tnfrsf12a*. Bar plots shown are mean ± SD (LD, n = 14; ADV, n = 16). P values were calculated using two-sided Wilcoxon rank-sum test.

To further investigate the paradoxical observation of increased expression of myogenic regulators alongside reduced grip strength and muscle weight, gene set enrichment analysis (GSEA) was performed using the Hallmark gene set from the Molecular Signature Database (MSigDB) (Castanza et al., 2023). The most significantly enriched gene set was the TNFα signaling pathway via NF-κB (Figure 3A; Supplementary Table 3), which is interesting because tumor necrosis factor (TNFα) is known to be associated with sarcopenia with its ability to promote protein catabolism, muscle degeneration and muscle atrophy (Sishi and Engelbrecht, 2011; Zhou et al., 2016). Therefore, we next examined the expression of genes in the TNFα signaling pathway. Whereas *Tnf* expression was not significantly increased in muscle tissues from the ADV group compared to the LD, *Tnfsf12*, encoding TNF-like Weak Inducer of Apoptosis (TWEAK), was significantly increased in the ADV group, along with its receptor Fn14 encoded by *Tnfrsf12a* (Figure 3B; Supplementary Figure 3A). This is interesting because TWEAK is a pro-inflammatory cytokine of the TNF superfamily, and recognized as a key mediator of muscle wasting during aging (Dogra et al., 2007; Tajrishi et al., 2014a). In addition, the expressions of *Myod1,* NF-κB-related transcription factors (*Rela*, *Nfkb1*, and *Nfkb2*), and unfolded protein response-related genes (*Atf4* and *Ddit3*) involved in the repression of protein synthesis were significantly higher under the ADV condition (Figure 2C; Supplementary Figure 3B). These genes are known downstream targets of the TWEAK/Fn14 signaling pathway (Tomaz da Silva et al., 2025; Zhou et al., 2016), suggesting that CJL activates the TWEAK/Fn14 signaling pathway. Collectively, the observed transcriptional changes in our CJL mouse model recapitulates key molecular features known to be associated with sarcopenia.

### 3.4 CJL causes muscle atrophy despite elevated histological markers for muscle regeneration and remodeling

**FIGURE 4:**
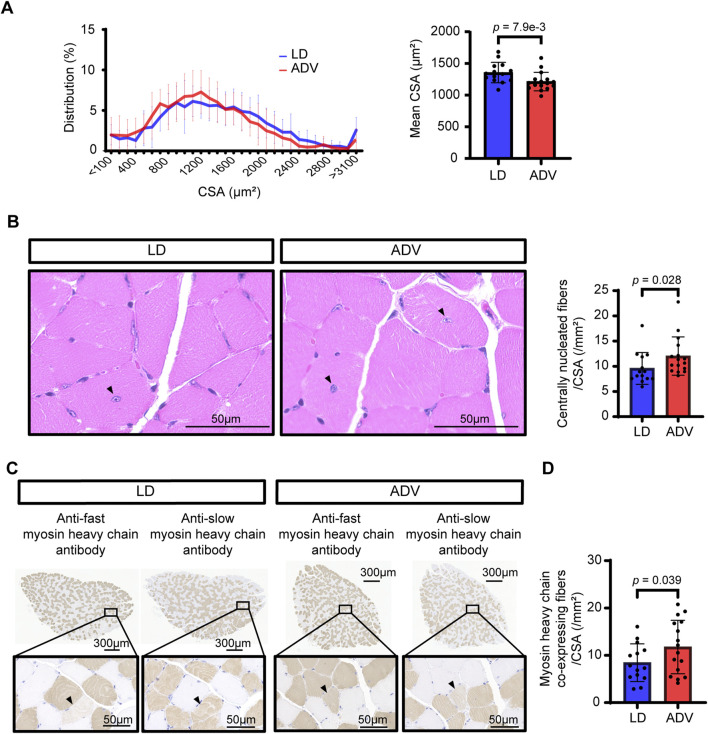
CJL causes muscle atrophy despite elevated histological markers for muscle regeneration and remodeling. **(A)** Histogram of gastrocnemius muscle fiber cross-sectional area (CSA). The distribution and mean CSA of muscle fiber are shown for the LD and ADV groups. The x-axis represents the CSA (µm^2^), and the y-axis indicates the relative frequency of muscle fibers within each bin of the histogram. P values were calculated using two-sided Wilcoxon rank-sum test. **(B)** Representative images of centrally nucleated fibers in gastrocnemius muscle from LD and ADV groups, and quantification of centrally nucleated fibers normalized to muscle CSA (mm^2^). Black arrow heads indicate centrally nucleated fibers. Scale bars: 50 µm for magnified regions. P values were calculated using two-sided Wilcoxon rank-sum test. **(C)** Representative immunohistochemical images of soleus muscle in LD and ADV groups stained with anti-fast myosin heavy chain (MHC) antibody and anti-slow MHC antibody. Black arrow heads indicate MHC co-expressing fibers, marked by overlapping staining of fast and slow MHC. Scale bars: 50 µm for magnified regions, 300 µm for overall views. **(D)** Quantification of MHC co-expressing fibers normalized to muscle CSA (mm^2^). Bar plots shown are mean ± SD (LD, n = 14; ADV, n = 16). P values were calculated using one-sided unpaired t-test.

To investigate the effects of CJL on skeletal muscle morphology, we examined the CSA of gastrocnemius muscle fiber using HE staining. The mean CSA of the muscle fiber was significantly smaller in the ADV group compared to the LD group (Figure 4A), indicating circadian misalignment induces muscle atrophy. These results are consistent with the observed reductions in grip strength and muscle weight, as well as with RNA-seq-based predictions involving activation of the TWEAK/Fn14 signaling pathway, suggesting that our mouse model also recapitulates histopathological features of sarcopenia. To experimentally validate RNA-seq-predicted enhancement of regenerative activity, we next quantified centrally nucleated fibers, a well-established histological marker of regenerated myofibers known to increase following muscle injury or exercise (Meyer, 2018; Narita and Yorifuji, 1999; Soffe et al., 2016; Wernig et al., 1990). The number of centrally nucleated fibers was significantly elevated in the ADV group compared to the LD group (Figure 4B), suggesting an accumulation of regenerating myofibers under CJL. Notably, despite reduced wheel-running activity levels in the ADV group relative to the LD group (Supplementary Figure 2), the observed increase in centrally nucleated fibers indicates that CJL itself promotes muscle regeneration. To further assess increased remodeling process, we quantified MHC co-expressing fibers (Andersen et al., 1999; Snow et al., 2005; Yoshimura et al., 1998). Skeletal muscle is composed of fast and slow muscle fibers, which generally express either fast muscle- or slow muscle-specific MHC (Blaauw et al., 2013). However, some muscle fibers are known to co-express both MHC markers. The presence of fibers co-expressing fast and slow muscle-specific MHC isoforms has been observed in intermediate fiber-type transitions such as the regenerative process or reinnervation following denervation (Jerkovic et al., 1997; Launay et al., 2006; Marini et al., 1991; Snow et al., 2005; Yoshimura et al., 1998). Therefore, we used MHC co-expressing fibers for semiquantitative analysis as a marker of ongoing remodeling. Because soleus muscle is relatively rich in slow twitch (Type I) muscle fibers and is commonly used for histological analysis of MHC markers (Snow et al., 2005), we performed quantitative analysis of MHC co-expressing fibers in soleus muscle. Immunohistochemical staining of muscle tissue was performed using monoclonal anti-fast and anti-slow MHC antibodies, and as expected, most fibers expressed only one MHC isoform (Figure 4C). Importantly, the number of MHC co-expressing fibers, along with centrally nucleated fibers, was significantly increased under the ADV condition compared to the LD group (Figure 4D; Supplementary Figures 4A, B). These results support the notion that chronic circadian misalignment enhances regeneration and remodeling processes including fiber-type transitions in skeletal muscle. However, these processes appear insufficient to maintain muscle homeostasis, which ultimately contributes to progressive muscle atrophy.

## 4 Discussion

While it is well established that circadian clocks play important roles in both skeletal muscle function and aging physiology in general (Harfmann et al., 2015), how circadian rhythms influence sarcopenia remains poorly characterized. Our study demonstrates that environmental circadian misalignment contributes to the progression of sarcopenia. To date, few reports have provided evidence linking shift work with muscle dysfunction and sarcopenia (Choi et al., 2019), leaving open the important question of whether circadian rhythms constitute an actionable target for sarcopenia prevention. In human epidemiological studies, it is challenging to determine the direct impact of circadian misalignment on muscle health since they are influenced by many confounding factors, including genetic background, lifestyle, and dietary habits. To overcome this limitation, we conducted a 64-week functional study in genetically homogeneous wild-type C57BL/6J mice using an established CJL paradigm devoid of major confounding factors. We observed that ADV mice exhibited exacerbation of age-related reductions in grip strength and normalized muscle weight compared to young mice and LD mice. These results underscore the physiological importance of an environmentally aligned lifestyle to maintain muscle health and suggest that circadian rhythms should be considered as an important factor in the prevention of sarcopenia.

Importantly, unlike previous genetic models, our results show that prolonged light cycle perturbation, even in the absence of genetic predisposition, is sufficient to reduce grip strength and cause muscle atrophy. While circadian misalignment, known as a mismatch between the intrinsic circadian oscillation and the external cycle, is associated with reduced survival and systemic maladaptation, its impact on organ-specific, especially skeletal muscle, pathophysiology has remained poorly understood (Highkin and Hanson, 1954; Hurd and Ralph, 1998; Ouyang et al., 1998; Pittendrigh and Minis, 1972; Takasu et al., 2015). Previous studies showed that genetic disruption of core clock genes impairs muscle function. In particular, both global and muscle-specific *Bmal1* knockout mice display sarcopenia-like phenotypes (Fernández-Martínez et al., 2024; Kondratov et al., 2006). Conversely, muscle-specific *Bmal1* rescue restored muscle strength and glucose tolerance, but did not affect muscle mass or fiber size (Gutierrez-Monreal et al., 2024). Moreover, reconstitution of endogenous *Bmal1* only in the SCN or in skeletal muscle fails to fully reverse the muscle atrophy, and restoration of both clocks is required to prevent the decline in muscle function (Kumar et al., 2024). Whereas these observations underscore the need for the coordination of central and peripheral clocks to maintain muscle homeostasis, it remains challenging to disentangle the effects of circadian misalignment itself from those of genetic disruption. Considering our previous studies (Inokawa et al., 2020; Koike et al., 2024), the CJL paradigm represents a systemic circadian misalignment model, encompassing not only local effects within skeletal muscle but also multifaceted physiological consequences in other organs, such as the liver. Accordingly, our study provides compelling evidence that environmental circadian misalignment alone without genetic manipulation can drive sarcopenia, highlighting the importance of maintaining alignment between endogenous circadian rhythms and external environmental cycles.

Activation of the TWEAK/Fn14 signaling pathway has been shown to be activated in aged mice (Tajrishi et al., 2014a). Our study revealed that circadian misalignment activates the TWEAK/Fn14 signaling pathway relative to age-matched control mice, suggesting that chronic circadian misalignment exaggerates age-related molecular alterations. Consistently, the ADV group also exhibited phenotypic features commonly observed in sarcopenia, including reductions in grip strength and muscle weight, activation of the TWEAK/Fn14 pathway, and associated muscle atrophy. These findings suggest that chronic circadian misalignment promotes the pathophysiological processes underlying sarcopenia.

Progressive muscle degeneration in sarcopenia can be driven by both molecular abnormalities, such as decreased regeneration efficiency, chronic inflammation, and disrupted protein homeostasis, and systemic changes including degeneration of the neuromuscular junction and anabolic resistance (Damanti et al., 2025). Mammalian skeletal muscle is a stable tissue with minimal turnover under normal conditions, but severe injury can trigger rapid and extensive regeneration (Tedesco et al., 2010). Our transcriptomic analysis revealed upregulation of key myogenic regulatory factors including *Myod1, Myog,* and *Myf6,* suggesting activation of muscle repair or remodeling processes. Although circadian misalignment may contribute to muscle degeneration, detailed histological analysis of degenerative changes was not performed in this study. Therefore, how circadian misalignment induces muscle degeneration remains to be elucidated in future investigations.

Interestingly, circadian misalignment activated the muscle regeneration and remodeling processes, accompanied by higher amounts of centrally nucleated fibers and MHC co-expressing fibers, whereas the mean CSA of the muscle fiber was decreased. These histological features suggest that regeneration and remodeling processes are insufficient, thereby leading to muscle atrophy. Previous studies have shown that the TWEAK/Fn14 signaling pathway contributes to muscle regeneration by regulating myoblast differentiation and proliferation, but excessive or sustained activation impairs regeneration by hindering myogenic differentiation and fusion, and suppresses protein synthesis, and enhances muscle protein catabolism through NF-κB-driven activation of the ubiquitin-proteasome system (Dogra et al., 2006; Dogra et al., 2007; Enwere et al., 2012; Tajrishi et al., 2014b; Tomaz da Silva et al., 2025). In line with these findings, our analysis revealed upregulation of the unfolded protein response–related genes *Atf4* and *Ddit3*, which are known to suppress protein synthesis (Harding et al., 2000; Tomaz da Silva et al., 2025). Furthermore, the intrinsic muscle clock is known to play a cell-autonomous role in coordinating anabolism and catabolism. The muscle clock contributes to the coordination of daily metabolic rhythms by enhancing lipid storage and restricting proteolytic activity before the active phase, and regulates the ubiquitin–proteasome system (UPS) and autophagy (Dyar et al., 2018; Kelu and Hughes, 2025). Under the CJL condition, it is plausible that activation of TWEAK/Fn14 signaling may impair myogenic differentiation or maturation, contributing to incomplete regeneration or remodeling. In addition, disruption of the intrinsic muscle clock, which normally maintains the balance between protein synthesis and degradation, may ultimately accelerate the progression of muscle atrophy and functional decline observed in ADV mice.

## 5 Conclusion

In conclusion, we show that circadian rhythm misalignment in the CJL paradigm aggravates the progression of sarcopenia. Although this study has several limitations: first, as all experiments were performed exclusively in male mice, future studies in females are needed to clarify potential sex differences in circadian and muscular physiology; second, although our data implicate activation of the TWEAK/Fn14 pathway, future studies incorporating detailed analyses of inflammation, as well as pharmacological inhibition or genetic manipulation, are expected to provide deeper mechanistic insight and clarify the causal relationship between aging, circadian misalignment, and muscle atrophy; and third, the analyses did not resolve which specific compartments or cell populations within skeletal muscle (e.g., fiber types, satellite cells, or neuromuscular junctions) are most affected by circadian misalignment. Nevertheless, our findings highlight the importance of circadian rhythms as an actionable target in sarcopenia prevention strategies.

## Data Availability

Data are available in the paper and/or the Supplementary Materials. The raw and processed RNAseq data reported in this paper are made publicly available via National Center for Biotechnology Information’s Gene Expression Omnibus (GEO) accession number GSE296198. Any additional information required to reanalyze the data reported in this paper is available from the corresponding author upon reasonable request.
