# Supplementary material for: Chronic circadian misalignment accelerates sarcopenia progression in mice
Source: Front Physiol. 2025 Nov 13;16:1686942. doi: 10.3389/fphys.2025.1686942 (PMC12658988; doi:10.3389/fphys.2025.1686942)
Supplement: Supplementary file 3 [file DataSheet1.pdf]

Supplementary Material

Chronic circadian misalignment accelerates sarcopenia progression in mice

Takashi Seya, Nobuya Koike, Naoki Okubo, Yasuhiro Umemura, Yoshiki Tsuchiya, Kazuya Yabumoto, Yasuhiro Endo, Kanako Iinuma, Akiyo Kakibuchi, Akira Sugimoto, Kenji Takahashi, Seung-Hee Yoo, Zheng Chen and Kazuhiro Yagita*

*** Correspondence:**

Kazuhiro Yagita

kyagita@koto.kpu-m.ac.jp

1 Supplementary Figures

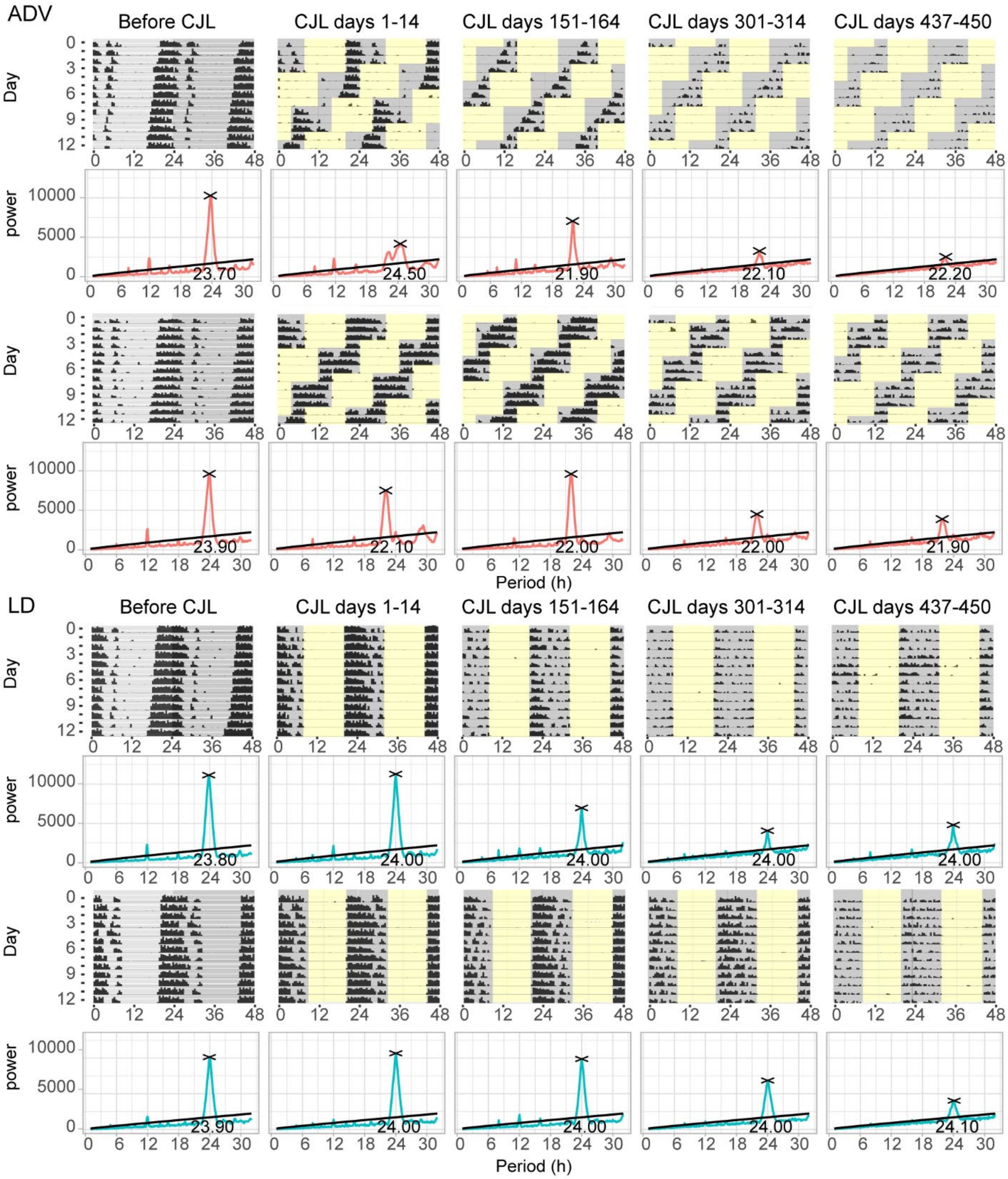

Supplementary Figure 1. Representative actograms and chi-square periodogram of wheel-running activities in LD and ADV groups. The chi-square periodogram was calculated using two weeks during baseline constant darkness (before CJL), and at 1–14, 151–164, 301–314, and 437–450 days in CJL. The oblique line in the periodogram indicates the significance level of $\alpha = 0.001$. The highest peak value above the line is indicated. Representative data from two animals per group are shown, selected from LD ($n = 14$) and ADV ($n = 16$) mice.

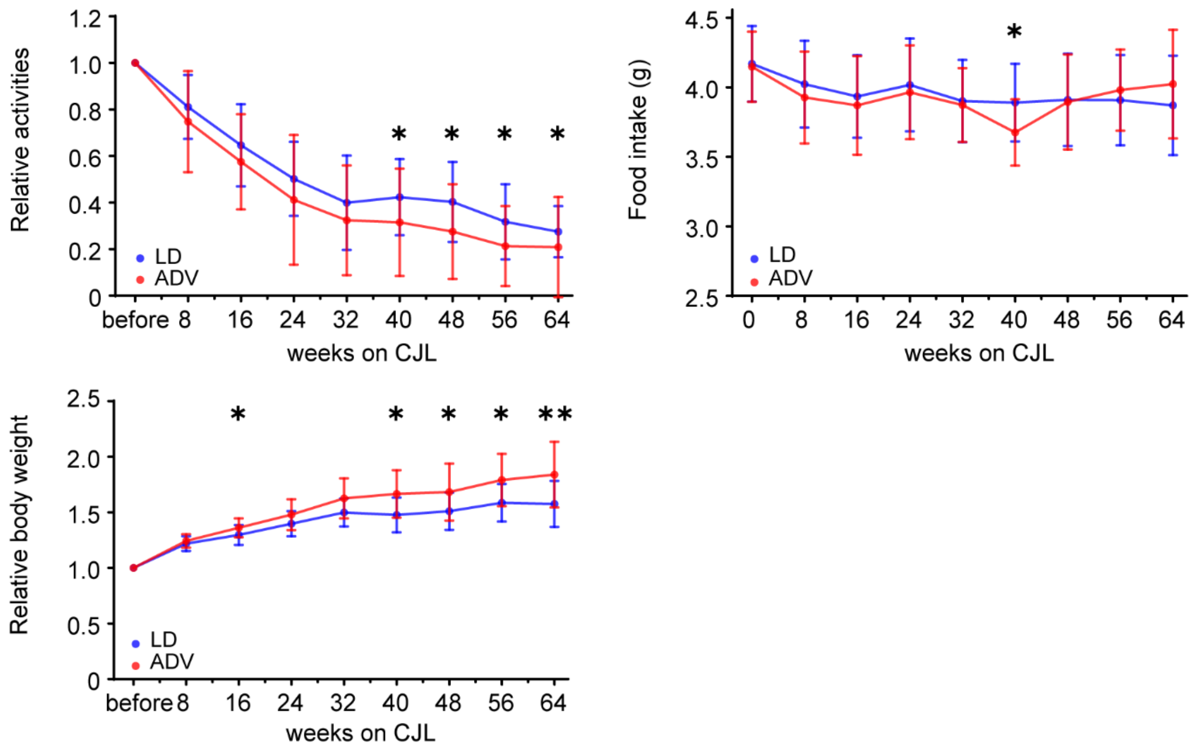

Supplementary Figure 2. The mouse physiology induced by CJL. Longitudinal changes of relative activities, food intake, and relative body weight, induced by CJL in LD and ADV groups. The relative activities were calculated as mean every 4 weeks during CJL and shown as the relative to mean value during the 3-week period before starting CJL. The body weights shown as relative values compared to 4 weeks before starting CJL. Data shown are mean ± SD (LD, n=14; ADV, n=16). P values were calculated using two-sided Wilcoxon rank-sum test (*p < 0.05, **p < 0.01).

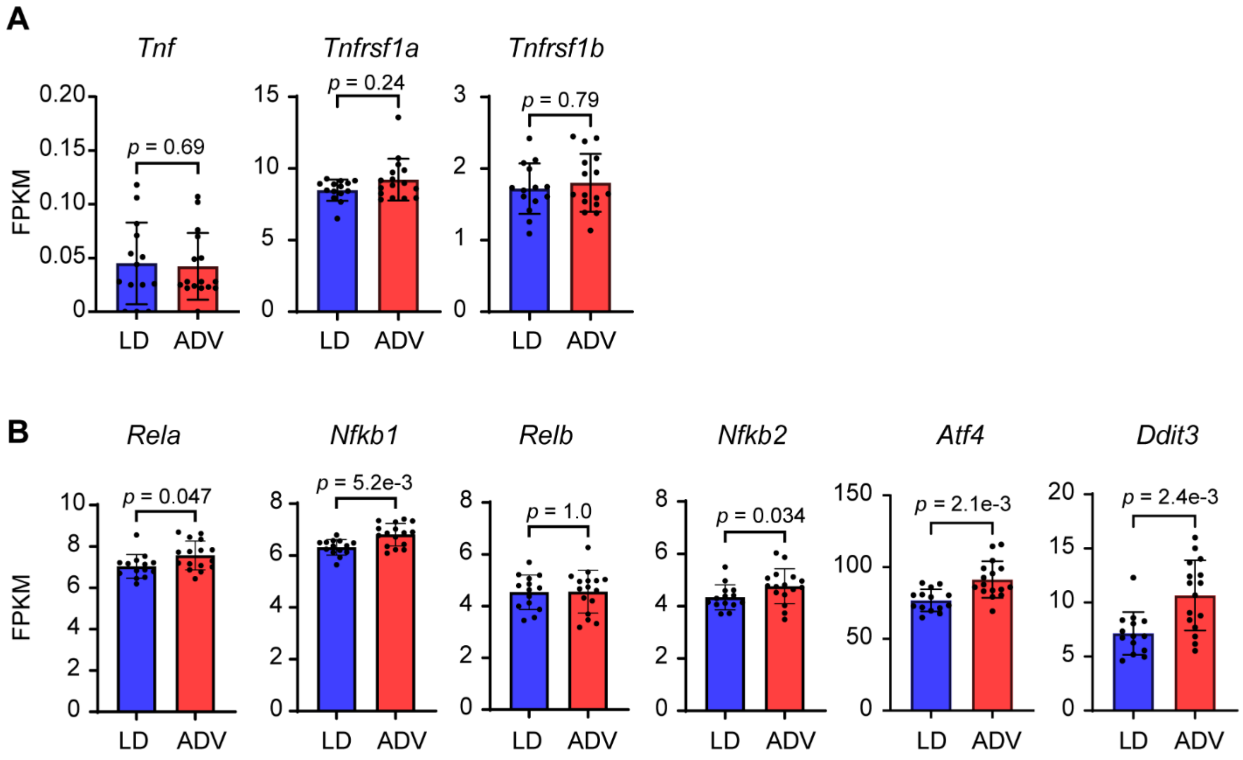

Supplementary Figure 3. Expression levels of TNFα and TWEAK/Fn14 signaling pathway related genes. (**A**) Expression levels of *Tnf* and TNF receptor genes. Comparison of FPKM expression levels of *Tnf*, *Tnfrsf1a*, and *Tnfrsf1b* between LD and ADV groups. (**B**) Comparison of FPKM expression levels of *Rela*, *Nfkb1*, *Relb*, *Nfkb2* between LD and ADV groups. Bar plots shown are mean ± SD (LD, n=14; ADV, n=16). P values were calculated using a two-sided Wilcoxon rank-sum test.

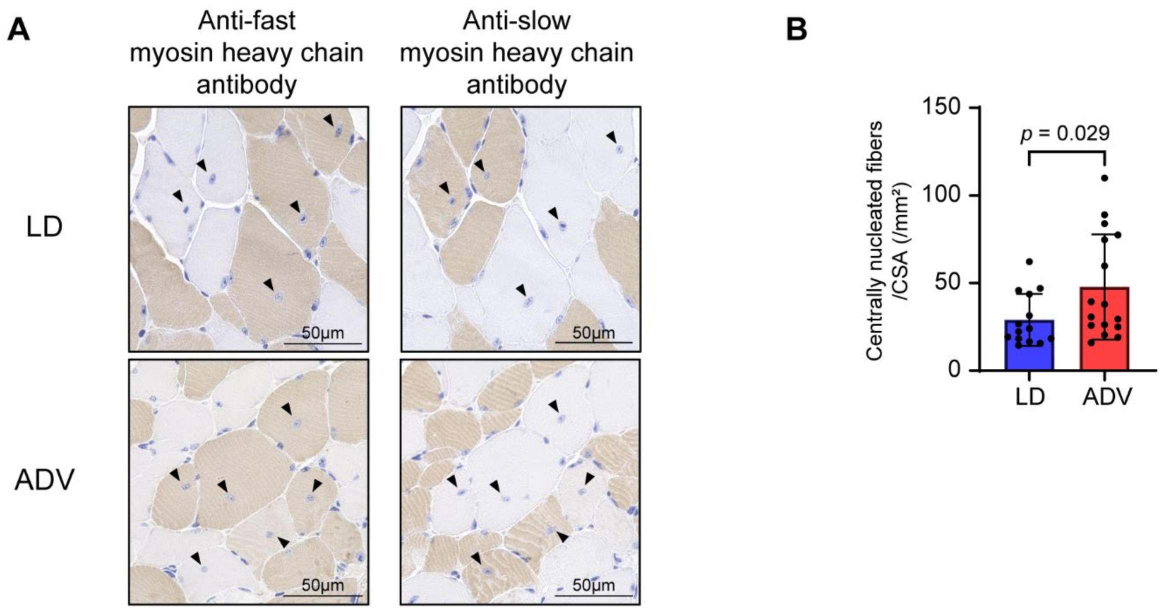

Supplementary Figure 4. Quantification of centrally nucleated fibers in soleus muscle. (**A**) Representative immunohistochemical images of centrally nucleated fibers in soleus muscle from LD and ADV groups stained with anti-fast MHC antibody anti-slow MHC antibody. Black arrowheads indicate centrally nucleated fibers. (**B**) Quantification of centrally nucleated fibers normalized to muscle CSA (mm^2). Bar plots shown are mean ± SD (LD, n=14; ADV, n=16). P values were calculated using one-sided Wilcoxon rank-sum test.

2 Supplementary Tables

Supplementary Table 1. List of DEGs in LD and ADV groups.

Supplementary Table 2. GO enrichment analyses of upregulated and downregulated DEGs.

Supplementary Table 3. Gene set enrichment analysis of the gastrocnemius muscle genes.
